# Sustainable Management of Organic Wastes in Sharjah, UAE through Co-Composting

**DOI:** 10.3390/mps3040076

**Published:** 2020-11-05

**Authors:** Md Maruf Mortula, Aqeel Ahmed, Kazi Parvez Fattah, Ghina Zannerni, Syed A. Shah, Ahmed M. Sharaby

**Affiliations:** 1 Department of Civil Engineering, American University of Sharjah, Sharjah 26666, UAE; mmortula@aus.edu (M.M.M.); aqeelahmed@aus.edu (A.A.); gzannerni@aus.edu (G.Z.); b00029643@alumni.aus.edu (A.M.S.); 2 Compost Plant, Sharjah Municipality, Sharjah 26666, UAE; syedshah356@hotmail.com

**Keywords:** co-composting, sewage sludge, green waste, food waste, organic waste, sustainable waste management, nutrient recovery

## Abstract

Daily human activities and vast green areas produce substantial amounts of organic wastes that end up in landfills with minimal treatment. In addition to the problems associated with landfills, disposal through this method is unsustainable in the long run and does not allow recovering materials from the waste. This paper focuses on the co-composting of different organic wastes produced in The Emirate of Sharjah, United Arab Emirates (UAE), to optimize mixing proportions of three different kinds of wastes—sewage sludge (SS), green waste (GW), and food waste (FW). All three organic wastes were analyzed to determine their chemical composition and the mixing proportions. Ten different mixing proportions as a function of carbon:nitrogen (C:N ratios) were determined and mixed in a NatureMill composter. Compost samples were tested for pH, salinity, conductivity, moisture content, organic matter, organic carbon, phosphorus, total nitrogen, and final C:N ratio after 6 weeks. Results indicate that a period of 5–6 weeks is sufficient for the compost to stabilize. The varying mixing proportions produced a good-quality compost (C:N up to 20:1) with high nutrient content. The study reaffirms that co-composting can be a potential sustainable organic waste management option for the United Arab Emirates.

## 1. Introduction

The proper and efficient management of different organic wastes produced is a large undertaking for any city. Rapid population growth and urbanization have accelerated the production of different types of wastes which has brought about the need for increased land requirement to dispose of these wastes. Often, these waste disposal sites or landfills are located far from the city or waste generation sites. The collection, transport, and disposal of the vast amount of wastes in landfills are a major environmental concern worldwide. The negative impacts of these wastes have restricted the potential growth of modern cities. Developing cities and countries witnessing a rapid expansion without a complex waste management system have often found handling of these wastes a burden to their development. 

There are different methods employed worldwide to dispose of municipal waste, from low-cost open dumps to more complicated and expensive processes such as pyrolysis [1]. Some processes are less sustainable and do not allow for reuse, recycling, or material recovery options. Municipal waste is usually discarded in landfills where it undergoes a combination of biotic and abiotic conditions producing methane, a gas 23 times higher in global warming potential than carbon dioxide [2]. With reducing land availability for landfills around cities, the waste is often incinerated to reduce waste volume by as much as 90%, with resulting waste products such as ash, metals, and glass sent to the landfill [3,4]. Although an added advantage of incineration is the production of energy that has the potential to reduce around 2 Mt of CO_2_ eq per year when compared to energy production by coal, the process does harm the environment [5]. According to Farrell and Jones [6], recycling and composting of organic matter introduce lesser negative impacts on the environment compared to regular disposal into landfills and incineration and, hence, composting organic waste is considered a sustainable waste management approach. Composting reduces soil pollution, carbon emission, and operational cost when compared to landfilling [7], while its applications to soil are able to decrease the negative impacts of urbanization on soil properties and enhance the environmental services associated with green infrastructure, such as nutrient cycling and efficiency, carbon storage, soil water capacity, and microorganism biocontrol [8]. The process is environmentally friendly as it involves a biological degradation of the organic matter in wastes under a controlled aerobic process [8,9]. In the process of composting, organic waste is transformed, by certain types of microorganisms, into more biologically stabilized products such as organic fertilizers or soil conditioners that are used to increase the level of nutrients in the soil [10]. In Europe, almost 47% of the waste was composted in 2016 [11], whereas, in the Middle Eastern countries, landfill disposal is the most widely practiced method for domestic solid waste management due to available desert land [12,13]. The quality of the generated compost can be measured by its maturity and stability. The maturity and stability of final compost are impacted by many factors. Therefore, optimizing the parameters of the compost such as transformation time, thermal phases, C/N ratio, and moisture, is the way to optimize the composting process. Transformation time affects the process in many ways; a short time will cause inadequate degradation of the organic matters with high molecular weight, while a long transformation time may lead to loss of nutrients [10]. According to Zhu [14], a proper C:N ratio for raw materials is around 20. The moisture content can be more than 85% in vegetable wastes. A pilot-scale study conducted by Bian et al. [10], where agricultural wastes with multi-components such as vegetable leaves, chicken manure, and rice husks were composted, showed that the optimal parameters for composting were a temperature of 75 ± 5 °C and composting time of 18 h. Furthermore, rice husks were found to maintain their original morphology and remain as a conditioning agent during composting. 

The composting process of sewage sludge was found to increase the degradation and minimize the amount of pharmaceuticals like fluoroquinolones, heavy metals, and pathogens in the urban sewage sludge [15,16]. Bożym and Siemiątkowski [17] found that the maturation of sewage sludge compost affects the final product properties such as the amount of organic matter, concentration of total organic carbon, nutrients, and heavy metals. Moreover, the environmental risks of the use of raw sewage sludge are reduced when converted to sludge compost. The sewage sludge with tree pruning compost obtained by Moretti et al. [16] was found to have a reduced risk of nitrate leaching through immobilizing mineral nitrogen into a more stabilized form. However, there are certain drawbacks in the use of sewage sludge in composting such as time consumption and decreased concentrations of nutrients. In this situation, inorganic nutrients may be added in the composting process in order to meet the plant requirements [15]. Agricultural and green waste treatment processes are also becoming of great concern due to the production of large amounts of wastes, particularly in rural areas. Converting organic waste such as plant residue, livestock manure, and sewage sludge into a compost either in combination or separately produces soil amendment that is biologically stabilized and able to provide a higher concentration of available nutrients compared to inorganic fertilizers. However, composting various organic waste, termed as co-composting, was proven to be more effective since it enhances many biogeochemical processes that are microbially mediated and lowers the loss of nutrients during composting. Good-quality compost requires optimizing the proportions of different types of organic waste in compost to obtain the desired characteristics. There have been many studies conducted on co-composting. Mantovi et al. [18] used a co-compost of combined solid sewage sludge with wheat straw and reported a very high increase in the availability of nutrients such as nitrogen and phosphorus to wheat and sugar beet crops. Roca-Pérez et al. [19] found a significant impact of a co-compost of sewage sludge combined with rice straw on the properties of soil and on the growth of barley plants. However, it was found that higher application rates of sewage sludge in the co-compost led to a higher metal content (Cd, Pb) in the plant. Therefore, it was recommended that lower application rates of sewage sludge in the co-compost are more useful for plant crops. In their study, Rehman and Qayyum [15] prepared a co-compost from several combinations of sewage sludge, cow dung, and rock phosphate and found that the co-compost of 25% sewage sludge, 25% of manure, and 50% of rock phosphate was a sufficient, rich, and sustainable fertilizer for rice-wheat plants. Cofie et al. [20] reported that a combination ratio of 2:1 of market waste to fecal sludge led to a C:N ratio of 13. Kumar et al. [21] combined food waste and green waste to achieve a reduction of total volatile solid (TVS) by 33% in 12 days, at a moisture content of 60% and C:N ratio of 19.6. Additionally, Zhang et al. [22] conducted a study on the characteristics of sewage sludge and green waste in co-composting. They found that the combination of sewage sludge and green waste (with a weight ratio of 8:1) led to an increase in C:N ratio from 15.2 to 22.8. Despite the availability of several studies on co-composting, the focus of these studies has been on the combination of only two organic wastes in co-composting at the same time. However, studies combining more than two types of wastes are limited. 

In the United Arab Emirates (UAE), despite municipal solid waste comprises 40% of organic waste [23], primarily the sludge generated in wastewater treatment plants used for soil enhancement through composting with green waste only [24]. The majority of the food waste is sent to landfills. In the city of Sharjah, UAE, sewage sludge (SS), green waste (GW), and food waste (FW) are the most commonly produced organic wastes. For proper waste management in Sharjah, it is important to have a form of composting process using all of these types of wastes. Currently, the compost plant uses SS and GW for co-composting practice. However, preliminary studies conducted by the research group indicated the potential for effective composting [25,26,27]. The studies focused on bench-scale experiments conducted on the different types of waste, and the results indicated the corresponding compost quality to be within the limit set by the local regulatory bodies.

The objective of this study was to assess the characteristics of generated SS, FW, and GW and to determine appropriate mixing proportions for achieving good-quality compost. Mixing proportions were created with the intention of providing sustainable waste management by considering generation rates of the different types of organic wastes produced in the city. Laboratory-based tests were conducted to assess different characteristics of these wastes. Appropriate mixing proportions were identified on the basis of the amount of solid waste production in the city of Sharjah and a target C:N ratio of 20:1. Laboratory-based experiments were conducted on a bench-scale set-up to investigate the ability of these potential mixing proportions to achieve good-quality compost. The purpose of this paper was to show the applicability of co-composting as a sustainable solution to organic waste management in Sharjah, UAE.

## 2. Materials and Methods

### 2.1. Materials

Three types of waste, generated in Sharjah, UAE were used in the study. Food waste (FW) was collected from food outlets on the American University of Sharjah campus in Sharjah, UAE. The bulk of the food waste consisted of cooked food that was discarded at the end of the service day and kept in chillers overnight to be collected in the morning. Animal protein and sources of carbohydrate such as rice and noodles formed the majority of the food waste. Some protein sources (such as bones) were chunky and were bigger than 100 mm in the smallest dimension. 

Air-dried belt-pressed sewage sludge (SS) was collected from a secondary treatment facility in Sharjah. The SS is shipped daily to the Sharjah Compost Plant run by Sharjah Municipality. The material is black in color and heavy with a gelatinous texture and strong ammonia smell. It is the largest volume of organic waste produced within the municipality of Sharjah. Green waste (GW) from landscaping operations was collected from Sharjah Municipality. The GW was composed of leaves, twigs, and branches of varying sizes. First, the GW was left for two weeks to dry and then shredded to smaller chunks for composting operations. The majority of the green waste comprised dry tree leaves and small pieces of branches with diameters no bigger than 1 cm. The volume of GW was relatively smaller than that of the SS. The compost facility in Sharjah Municipality uses a mixture of SS and GW and produces a compost with C/N ratio less than 20. FW was not used within the compost mixture.

Characterization of different wastes was conducted using standard testing procedures. Tests were performed according to the Test Method for the Examination of Composting and Compost (TMECC) [28]. The obtained results are presented in Table 1.

### 2.2. Experimental Set-Up

**Figure 1:**
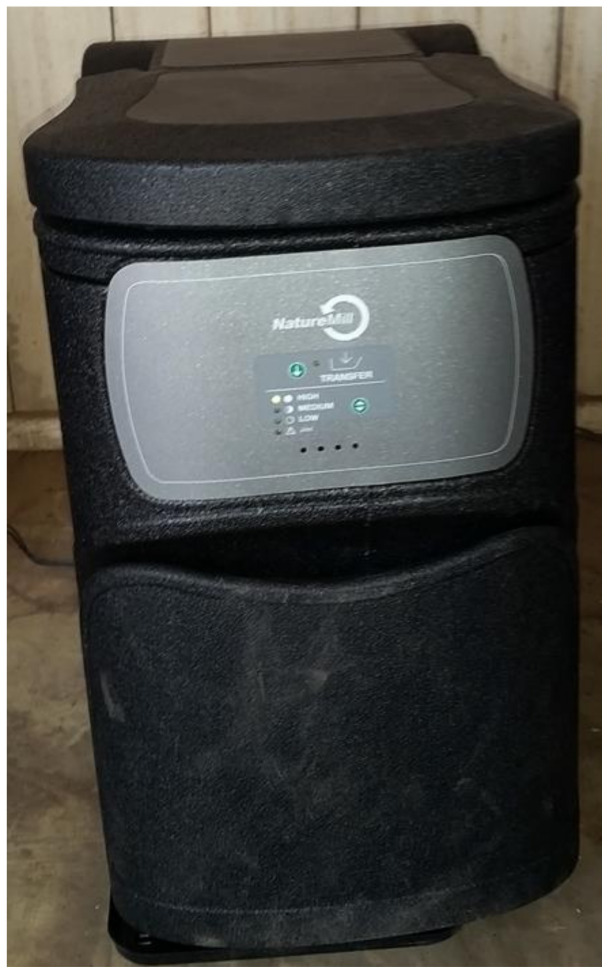
NatureMill composter.

Naturemill, a commercial domestic composter, was used for the bench-scale experimental set-up (Figure 1). It has two vertical chambers. The top opens to the first chamber where waste is discarded for mixing and homogenizing by a mechanical bar. The composter constantly aerates the compost, which is recommended to increase the total nitrogen content in the final compost [29]. Slanted flappers at the bottom of the first chamber allow small enough digested particles to pass through the openings into the second chamber to be collected. The smooth interior and minimized sharp corners help prevent accumulation and sticking. Naturemill controls the temperature up to 60 °C and the moisture of the composting process by keeping it unaffected by outside atmospheric conditions. It is also fitted with an activated carbon filter to minimize odors. The composter is capable of accommodating at least 10 L samples. Details of the composter can be found in [30].

### 2.3. Assessment of Mixing Proportion

The mixing of SS, GW, and FW was based on the proportions of SS, GW, and FW generated in the Sharjah Municipality to develop a sustainable waste management and the desired C:N ratio. Sharjah Municipality requires the C:N ratio to be less than 20, primarily due to the climatic conditions. In consideration of all these criteria, the optimum C:N ratio used for this study was around 15–20:1. The mixing ratios for the experiments were determined on the basis of initial C:N ratios and calculated using Equation (1) according to the characteristics of the different types of waste (Table 1) and mixing proportions. Table 2 shows the mixing proportions and the calculated C:N ratios. One of the experiments was planned to contain no FW (control experiment) so as to mimic current practices in the compost plant in the Sharjah Municipality.

(1) $$Final\;C:N=\;\left(R_{SS}\times \;W_{SS}\right)\;+\;\left(R_{GW}\times \;W_{GW}\right)\;+\;\left(R_{FW}\times \;W_{FW}\right),$$

where final C:N is the cumulative C:N ratio of SS, GW, and FW. R is the C:N ratio of each waste type—SS, GW, or FW. W is the proportion of each type of waste in the final weight of waste. The dry weights were used with appropriate moisture content and volume of the waste.

### 2.4. Experimental Approach

Nine experimental set-ups were placed in the Sharjah Compost Plant in a shed in a breezy area. Each set-up was filled with one combination of mixing proportions of SS, GW, and FW (Table 2). GW was placed first, and then the wetter ingredients were added to make use of gravity in order to evenly distribute the moisture. Once the different wastes were mixed, the set-up was then turned on to rotate the mixing lever and start the aeration and heating systems. A regular weekly monitoring was conducted to determine the extent of decomposition of the mix. Mixing proportions 4, 6, 7, and 8 were monitored for temperature on a daily basis for understanding the kinetic behavior. Proportion 8 was selected as a blank sample (without food waste). 

After the anticipated period of incubation, the resulting compost was then collected in sample bags, each sample weighing at least 500 g, and tested immediately or stored in a plastic container inside the refrigerator following standard methods to be tested later.

### 2.5. Analytical Methods

Samples were pulverized using a mortar and pestle prior to all tests. Standard analytical methods were used to test the characteristics of the waste samples and the final compost samples. Temperature was monitored during the composting process using an electronic probe. The characteristics investigated were pH levels, moisture content, salinity, organic matter at 550 °C, organic carbon, C:N ratio, total nitrogen, phosphorus, and color. Tests were performed according to the Test Method for the Examination of Composting and Compost (TMECC) [28]. 

## 3. Results and Discussion

### 3.1. Effect of Mixing Proportion on Compost Characteristics

#### 3.1.1. Temperature 

**Figure 2:**
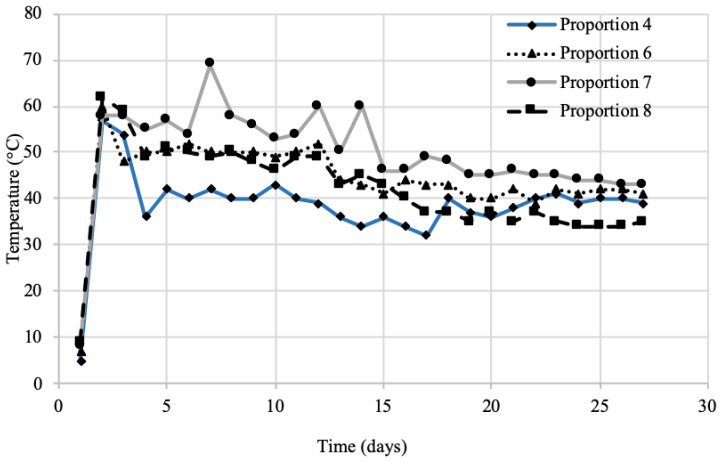
Temperatures of compost in the first month.

Figure 2 illustrates the temperature profiles during the composting process for four different mixing proportions. Temperatures rose immediately (2 days) after mixing reaching latency (>55 °C) [22], confirming that the presence of pathogens was minimized with a probability of more thermophilic bacteria. The temperatures approached the mesophilic regions (35 °C) toward the end of the first month. Mixing proportion 7 showed a high temperature for a longer period in comparison to the other compositions; this high temperature is beneficial during composting as it assists in the composting process and in eliminating harmful pathogenic microorganisms [3]. This, higher temperature profile could be due to the high C/N ratio that affected biological degradation of the waste mixture. 

#### 3.1.2. Physical Characteristics

The compost from all set-ups was ready for curing at the 6th week from the day it was combined. The fibrous and leafy part of green waste (GW) was hardly visible. However, the compost passed through the slits at the bottom of the composter. The almost dry to mildly wet compost obtained was dark brown in color and contained small fibers to coarse pieces of wood. The compost smell was similar to wet earth with a very a faint hint of ammonia, and the yield was visibly lower in weight and quantity than the original mixed material. 

#### 3.1.3. pH

The pH levels at the beginning of the compost are crucial to allow the natural occurring microorganisms to flourish in the early stages of the decomposition process. Organic acids are later produced due to the breakdown of organic material; however, the acidic conditions are more favorable for microorganisms. The final pH of a mature compost ranges between 6–8 in the neutral range [31]. The final pH values of Composts 1 through 9 were 6.7, 8.5, 8.2, 6.6, 6.8, 8.0, 7.7, 8.3, and 7.7, respectively. The test results only show the final obtained pH level of the resulting compost to determine if the quality of the compost produced from the SS, GW, and FW was satisfactory for use. With pH values near neutral or basic, these composts are suitable for low-pH soils. 

#### 3.1.4. Conductivity and Salinity

**Figure 3:**
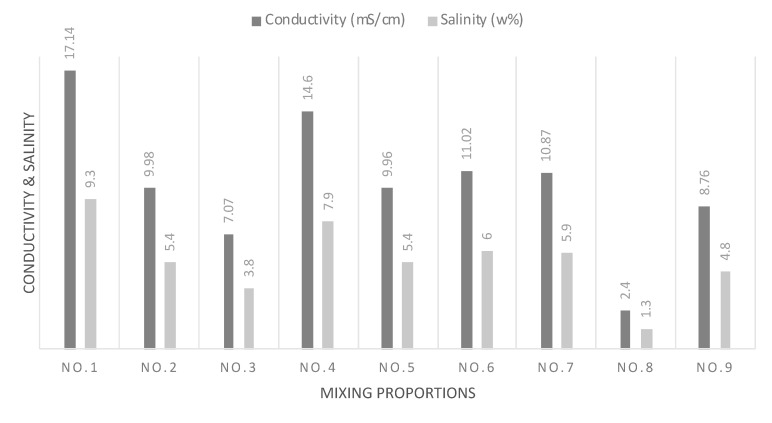
Conductivity and salinity of the compost.

Figure 3 shows the results for conductivity and salinity of the final compost. High conductivity was expected as GW and FW were used as main ingredients of the compost. In UAE, desalinated water with high salinity is used for the growth of plants. Eventually, it creates high conductivity in the GW and FW. The results below confirmed the expected high salinity. On the other hand, for commercial use, the high salinity may be a concern due to the unwanted effects that salinity has on soil quality and groundwater. The high conductivity may also be due to the existence of other forms of minerals as a result of the breakdown of the organic matter, which is a good indicator of a well-matured compost that can offer nutrients to the plant of slow-dissolving minerals [32]. However, as most water bodies in UAE contain high salinity, the local plants are accustomed to high salinity levels. The control experiment using SS and GW only showed the lowest conductivity by a big margin in comparison to the other experiments. The conductivity test of the final compost is a measure of stability of the compost. A stable compost is expected to have gone through enough decomposition that the final conductivity is lower. The lower conductivity is due to the decay of organic acids [33].

#### 3.1.5. Organic Matter

**Figure 4:**
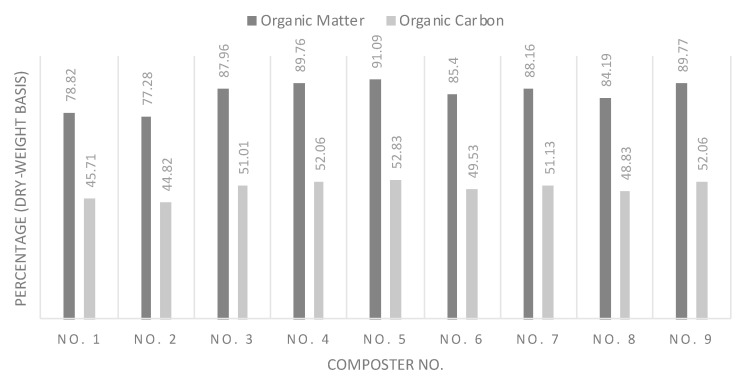
Organic matter (OM) (% of total dry mass) and organic carbon (OC) (% of organic matter) composition in compost.

Organic matter is shown in Figure 4. It is reported as a percentage of the weight of the compost, which is recommended to be above 40%. All composts satisfied the Sharjah Municipality Standards, as shown in Table 3.

It is worth noting that the control experiment showed one of the lowest organic matter percentages by weight. This could be due to the fact that sludge is decomposed faster than food waste in the composting process. The organic percentage by weight is also a good measurement of decay and stability. The results show that the dry mass in organic matter ranged between 63.58% and 75.63%. The three types of organic waste used as ingredients in the compost scored higher than 70% organic matter, with food waste reaching up to 93% organic matter. Thus, some level of decay must have happened to reach the resulting levels. Compost 8 with no FW was found to have one of the lowest percentages of organic matter, which proves that the content of food used in other compost raised the initial organic matter percentage.

#### 3.1.6. Organic Carbon

Organic carbon in the final compost was taken as a percentage of the organic matter. Figure 4 also shows the test results for organic carbon in the final compost. The organic carbon was later used to determine the C:N ratio of the resulting compost.

#### 3.1.7. C:N Ratio

The carbon-to-nitrogen (C:N) ratio is an important factor in producing and optimizing compost quality. Although the initial C:N ratio recommended is between 20–40 [34], a lower final C:N ratio is a good indicator of maturity of the compost. According to [21], co-composting of green waste and food waste can be done at a low C/N ratio. Operating at a low C/N ratio and a higher moisture content can assist in minimizing the use of bulking agents (materials which help regulate moisture content and attain optimum air space) in the co-composting process. For final compost, the carbon-to-nitrogen ratio is preferred to be 14 or less [35]. Watson et al. [36] concluded that, with a C:N ratio between 1 and 15, the nitrogen in the compost is released rapidly, which then allows for plants to take it up rapidly. In this study, the observed C:N ratios were 14.53, 14.55, 18.3, 22.67, 18.18, 10.53, 20.29, 17.71, and 16.85, respectively, for the nine set-ups, and only the results met the C:N preference of 15 or less. Compost 1 yielded a final C:N ratio of 14.53, while composts 2 and 6 yielded C:N ratios of 14.55 and 10.53, respectively. The other composts hovered around a 17 C:N ratio, whereas compost 4 had a value in the 20s. The increase in proportion of green waste increased the C:N ratios. The final C:N ratio acceptance border was used as a measure of decomposition of the organic matter of the compost. As the compost is digested, the carbon is converted to carbon dioxide due to biological processes within the microorganisms. As the population of the microorganisms peaks, the decay of the population releases the nitrogen that was a constituent of the microorganisms’ bodies. Therefore, it is expected for the final C:N to be less than the starting C:N ratio. 

#### 3.1.8. Total Nitrogen

**Figure 5:**
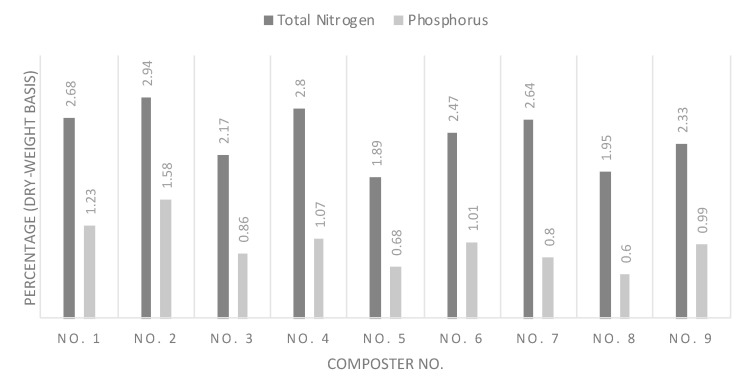
Total nitrogen and phosphorus (P_2_O_5_) in the final compost.

The composting process stabilizes the nitrogen through mineralization that introduces a slower and more constant source of nutrition for plants [37]. According to Figure 5, composter 2 showed the highest level of total nitrogen corresponding to a lower C:N ratio. Other compost C:N ratios ranged from 1.89 to 2.8. Nitrogen in the final compost in the majority of the yields seemed to be less than the mixing total nitrogen percentage. The loss could be due to the loss of ammonia in the thermophilic stage of the composting process. In addition, a low C:N ratio (lower than the optimum of 30) can result in anaerobic conditions, leading to nitrogen loss in the form of ammonia gas [3].

#### 3.1.9. Total Phosphorus

Figure 5 shows the phosphorus present in the final compost. Compost 2 again showed the highest level of phosphorus (1.58% on a dry-weight basis). Compost 2 produced higher levels of nutrients, i.e., nitrogen and phosphorus, in comparison to the blank set-up (compost 8). The increase in phosphorus is important because a considerable amount of phosphorus is lost due to erosion, especially in the hot and dry climate of UAE. It can be concluded that FW can enhance the quality of the compost as a fertilizer as long as it conforms with the upper limits of those elements in soil. Overall, all systems achieved higher phosphorus concentrations than the blank system. 

## 4. Conclusions

While co-composting of GW and SS is common, co-composting of SS, GW, and FW is uncommon in real practice, especially for commercial production or use. This study investigated the applicability of using the three wastes for composting as a means of providing a sustainable organic waste management option. The resulting compost was characterized with respect to the pH, salinity, and organic content, especially carbon, C:N, nitrogen, and phosphorus. After analyzing the three organic wastes and using the C:N ratio as a basis for mixing proportions, it was found that the three organic wastes produced good-quality compost in small scales within 5 weeks of mixing. The presence of food waste increased the operating temperature during the composting process compared to the proportion without food waste (proportion 8). There was an increase in the salinity and conductivity upon the addition of food waste. The presence of organic matter and carbon was not considerably affected among the different proportions. The calculated C:N ratio ranged from low (around 15:1) to medium (around 20:1) and still produced good-quality compost. The observed C:N ratio was mostly similar to the initial estimated ratios. The increase in proportions of green waste increased the C:N ratio. Compost 2 produced the compost with the highest nutrient content (both nitrogen and phosphorus) and, therefore, would provide the highest fertilizing capacity. The study showed that, while co-composting could be implemented as a sustainable organic waste management option for the city of Sharjah, it is important to determine the conditions and mixing proportions that will provide the best product. In the next stage, feasibility for larger scale co-compost production is being studied to further understand and enhance the process. 

## Figures and Tables

**Table 1:** Characterization of sewage sludge, green waste, and food waste used in the study.

	Sewage Sludge	Green Waste	Food Waste
pH	7.1	6.05	4.33
Conductivity, mS/cm	0.78	11.4	5.3
Salinity ^1^, %	0.4	6.5	2.8
Moisture content ^1^, %	80.8	7.9	58.9
Organic matter ^1^, %	72.1	70.8	93.7
Organic carbon ^1^, %	41.8	41.16	54.4
C:N ratio ^1^	7.45	37.0	15.7
Total nitrogen ^1^, %	5.6	1.1	3.5
Phosphorus as P_2_O_5_^1^, %	2.6	0.3	0.7
Density, g/L	215	34	194

^1^ by dry weight.

**Table 2:** Summary of dry weight-based proportions (as percentages) of mixed organic waste.

Mixture	Sewage Sludge	Green Waste	Food Waste	Total Waste (L)	C:N Ratio (*w/w*)
1	50	15	35	8.5	14.79
2	40	15	45	8.6	15.61
3	30	15	55	6.7	16.43
4	50	20	30	4.9	15.85
5	40	20	40	4.9	16.67
6	30	20	50	4.9	17.50
7	40	35	25	8.7	19.87
8	86	14	0	6.1	11.61
9	50	30	20	6.7	17.98

**Table 3:** Sharjah Municipality compost quality standards.

Parameter	Standard
pH	≤7.5
Conductivity, mS/cm	≤10
Salinity, %	<2%
Moisture content, %	<25%
Organic matter at 550 C, %	≥40%
Organic carbon, %	Around 54%
C:N Ratio	≤20:1
Total nitrogen, %	From less than 1% to 5%
Phosphorus as P_2_O_5_, %	NA
